# Scalar implicatures: working memory and a comparison with *only*

**DOI:** 10.3389/fpsyg.2013.00403

**Published:** 2013-07-18

**Authors:** Paul P. Marty, Emmanuel Chemla

**Affiliations:** ^1^MIT Department of Linguistics and Philosophy, Massachusetts Institute of TechnologyCambridge, MA, USA; ^2^Laboratoire de Sciences Cognitives et Psycholinguistique, Ecole Normale Supérieure, (CNRS, EHESS; DEC, ENS)Paris, France

**Keywords:** scalar implicatures, grammar, pragmatics, working memory, language processing

## Abstract

A Scalar Implicature (SI) arises when the use of a relatively weak sentence (e.g., ***some** politicians are corrupt*) implies the denial of an alternative, stronger sentence (e.g., *not **all** politicians are corrupt*). The cognitive effort associated with the processing of SIs involves central memory resources (De Neys and Schaeken, 2007; Dieussaert et al., 2011; Marty et al., 2013). The goal of this study is to locate this previous result within the current psycholinguistic debate, and to understand at which level of SI processing these resources are specifically involved. Using a dual-task approach, we show that (1) tapping participant's memory resources interferes with the derivation of SIs, whereas (2) it does *not* affect the interpretation of sentences involving similar competition mechanisms between a sentence and potential alternatives through the use of *only* (e.g., ***only some** politicians are corrupt*). We explain how these findings suggest that the central memory resources are not involved in the core process at the source of SIs, and discuss how this difference between SIs and *only* bears on recent linguistic debates on the division of labor between grammar and pragmatics.

## Introduction

A weak sentence with a set of alternatives normally implies the negation of the stronger alternatives. Consider for instance the following sentence:

(1) Some politicians are corrupt.

Semantically, the quantifier *some* encodes a weak, lower-bound meaning (i.e., *at least two*), which is logically consistent with *all*. Under its literal reading, the sentence in (1) is thus compatible with a situation in which *all* politicians are corrupt. Yet, hearers usually infer from an utterance of (1) that *not all* politicians are corrupt, as if *some* was attributed a strong, doubly bounded meaning (i.e., *some but not all*). Such inferences have been referred to as Scalar Implicatures (SIs). Descriptively, the informational contribution of (1) ends up having the following two components:

(2) a. Some (compatible with all) politicians are corrupt. (Literal Meaning)
       b. Not all politicians are corrupt.        (Scalar Implicature)

Various arguments suggest that the *not-all* SI in (2-b) is not delivered by the regular semantics and is not part of the literal meaning of (1). Like other kinds of conversational implicatures, SIs are cancelable and can be defeased in the presence of appropriate linguistic cues without any infelicity arising (3-a), while the literal meaning cannot (3-b):

(3) a. Some politicians are corrupt.   In fact, all of them are.
       b. Some politicians are corrupt. #In fact, none of them are.

According to the traditional view (Grice, 1975, 1989), SIs are derived from a pragmatic reasoning about speaker's communicative intentions. In the present case, the sentence in (1) is in competition with the minimally different sentence *All politicians are corrupt*. This alternative is entertained because of a more basic competition between the lexical items *some* and *all*, which belong to the same informational (or semantic) scale (i.e., 〈some, all〉), that is a set of alternates ordered on the basis of informational strength (Horn, 1972; Katzir, 2007 for a modern approach). The *all*-alternative is superficially comparable to the uttered sentence, in terms of structure or length for instance, but it is informationally stronger since *all Xs are Ys* asymmetrically entails *some Xs are Ys*. Assuming then that the speaker of (1) is trying to be cooperative and to say as much as she truthfully can that is relevant to the current purposes of the exchange (following Grice's maxim of Quantity), the fact that she did not utter the *all*-alternative gives hearers reasons to think that she was not in a position to deliver the additional information, plausibly because she believes that this stronger statement is false. The negation of the *all*-alternative corresponds to the SI given in (2-b)^1^.

## Scalar implicatures and *only*

We may describe SIs using a so-called *exhaustivity* operator, let us call it 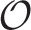, which would be very close in meaning to *only*. In a Gricean approach, this operator can be thought of as a pre-compilation of the operations driven by principles of rational cooperation and leading to the scalar inference (see Spector, 2003; van Rooij and Schulz, 2004). The idea is that the sentence in (4) with its SI is equivalent to the sentence with *only* in (5). Accordingly, (4) with its SI could be represented as in (6). Under this view, it is natural that 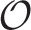 is optional and invisible, leaving room for the literal meaning to arise.

(4) Some politicians are corrupt.
(5) Only some politicians are corrupt.
(6) 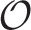
(some politicians are corrupt).

In recent years, following the so-called “grammatical” approach to SIs advocated by Landman (2000) and Chierchia (2004), it has been suggested that 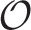 could be a plain grammatical operator (Fox, 2007; Chierchia et al., 2008, 2009). Under this alternative view, the sentences in (4) and (5) are very similar since the difference between them would simply be that the optional operator playing the role of *only* is covert in (4) [and made visible to the analyst's eyes as 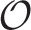 in (6)]. Even though the grammatical approach assumes that SIs are the result of a grammatical operation (as opposed to a pragmatic reasoning), it shares various components with the Gicean approach [see Chemla and Singh (submitted) for discussion]. In particular, the decision to apply 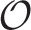 or not, or to stop at any point in the computation, is a pragmatic one under the two accounts. The derivation of alternatives is also identical in the two systems, involving both grammatical and pragmatic considerations.

It is beyond the scope of this paper to discuss the theoretical arguments for and against this grammatical move. Our results may ultimately contribute to the debate between Gricean and grammatical approaches to SIs (see discussion), but we will not commit ourselves to any particular view about the status of 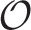 at this point. We simply rely on the insightful comparison between the implicit doubly bounded meaning of *some* and the explicit doubly bounded meaning of *only some* to narrow down different steps in the derivation of an SI as follows.

- The derivation of an SI involves the decision to apply 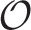. This decision is akin to a disambiguation decision, and it is of a pragmatic kind in both the grammatical and Gricean approach we sketched above. This decision may come at some cost in comparison with cases where the role of this operator is directly played by an overt lexical item such as *only*. This step constitutes therefore a noteworthy difference between (4) and (5).
- The derivation of an SI involves a comparison between a phrase and its alternatives. This second step may be identified as the stage at which the SI *per se* is computed. At this level, (4) and (5) are entirely similar: both 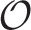 and *only* make it necessary to (a) identify a set of (otherwise implicit) alternatives (e.g., *all politicians are corrupt*), and (b) exclude the stronger alternatives (*not all politicians are corrupt*).

Importantly, the existence of these two steps is uncontroversial in the theoretical literature, beyond disagreements about the exact nature of the processes involved in implicature generation^2^. A summary of the *partial* analogy between the generation of SIs and the computation of the grammatical operator *only* is provided in Table 1.

**Table 1:** **Description of the steps involved in the doubly bounded interpretation associated with scalar items (e.g., *some*) and *only***.

		**SI**	**Only**
Step 1.	Decision to apply 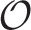	Yes	No
Step 2.	(a) Derivation of alternatives	Yes	Yes
	(b) Exclusion of alternatives	Yes	Yes

In the next section, we review previous psycholinguistic results about SI, and show how the analogy between 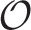 and *only* can help determine which properties of SIs are due to which step(s) of their derivation.

## The psycholinguistic processing of SIs

Psycholinguistic studies have initially focused on distinguishing two empirical models about how SIs are processed, a default model and a non-default model. As shown in Table 2, both models similarly assume that the different interpretations which scalar sentences can give rise to are generated through the following three processing stages. First, the grammatical composition rules determine the semantic contribution of scalar items to the literal interpretation of the sentences in which they occur (Stage 1). Next, this literal interpretation is enriched *via* implicature so as to yield an interpretation that includes the strengthened meaning of the target scalar items (Stage 2). The enriched interpretation can be finally retracted if there are linguistic—or extra-linguistic—reasons to cancel the previously triggered implicature (Stage 3).

**Table 2:** **Processing stages assumed by the default and non-default model**.

	**Processing stages**	**Interpretation (for *some*)**	**Accessibility**	
			**Default**	**Non-default**
1.	Semantic composition	LB: *some*	No	Yes
2.	Implicature generation	DB: *some (but not all)*	Yes	Yes
3.	Implicature cancelation	LB: *some (but not all)*	Yes	Yes

LB and DB refer to lower-bound and doubly bounded, respectively.

However, both models crucially differ with respect to the processing stages at which the literal interpretation is hypothesized to be accessible to comprehenders. According to the default model, Stage 2 is automatically applied, so that the output of Stage 1 cannot be accessed: implicatures arise upon the occurrence of an implicature trigger, independently of context. For instance, on hearing *Some politicians are corrupt*, the lower-bound meaning of *some* gets calculated but automatically strengthened by the negation of its stronger alternative *all*. As a result, accessing the literal interpretation requires an extra processing stage, the canceling of the *not-all* implicature. By contrast, the non-default model does not consider implicatures to be automatic processes. It assumes rather that Stage 1 is dissociated from Stage 2 in such a way that comprehenders can in principle (i.e., depending on contextual factors) directly access the literal interpretation as the output of Stage 1, with no need to go through Stages 2 and 3.

As Bott and Noveck (2004) noted (see also Noveck, 2001, Noveck and Posada, 2003), these two models make distinct, testable predictions with respect to the processing of the lower-bound (literal) and doubly bounded (with implicature) interpretations of scalar sentences. According to the default model, the lower-bound interpretation should require more processing effort than the doubly bounded interpretation, since hearers must derive and cancel the doubly bounded interpretation before accessing the lower-bound one. The non-default model does not predict the lower-bound interpretation to necessarily come at an additional processing cost, since hearers can access the lower-bound interpretation at Stage 1 before deriving the doubly bounded reading. These predictions have been experimentally tested for the last decade by means of various methodologies, including *inter alia* response time studies (Bott and Noveck, 2004), self-paced reading (Breheny et al., 2006; Bergen and Grodner, 2012), visual-world (Huang and Snedeker, 2009; Breheny et al., 2013), and “gumball” (Degen and Tanenhaus, 2011) paradigms. Results have been interpreted as providing convincing evidence against the default model of SI processing: in short, the doubly bounded reading is not accessed earlier than the lower-bound reading^3^.

### The time course of SIs: response time results

Bott and Noveck (2004) investigated in a truth-value judgment task the time course of the interpretation of scalar *Some*-sentences such as *Some elephants are mammals*, which are false with their *not-all* implicatures (since *all* elephants are mammals), but true under their literal meaning, i.e., without implicatures. Bott and Noveck found that participants (native speakers of French) took significantly more time to answer when generating doubly bounded than lower-bound interpretations, whether they were explicitly instructed to interpret *some* literally (i.e., *some or all*) or pragmatically (i.e., *some but not all*), or whether no specific instructions were given in this respect. They also found that limiting the time available for responding boosted the rate of lower-bound interpretations. These seminal results were interpreted as providing evidence for delayed implicature processing, falsifying the predictions made by the default model.

However, more recent studies (Grodner et al., 2010, Bale et al., 2011, Bott et al., 2012, *inter alia*) have discussed other possible factors that could account for this delay, and proposed alternative explanations for the longer processing times of implicatures relative to literal meanings. In particular, it has been suggested that longer response times could result from a greater difficulty in processing doubly bounded propositions, because these propositions, logically stronger than their lower-bound counterparts, may require more complex verification strategies (e.g., proving *some but not all Xs are Ys* requires finding one X that is Y *and* one other X that is not Y). As Bott et al. (2012) noticed, the greater informational complexity of doubly bounded propositions could increase response times independently of implicature calculation, which could blur the difference between the *derivation* of lower-bound and doubly bounded interpretations. It has been argued along these lines that proper controls with meanings equivalent to both possible interpretations (albeit not by means of implicatures) are needed to distinguish the contribution of implicature derivation and of more general proposition evaluation (Grodner et al., 2010; Bale et al., 2011).

Following this line of research, Bott et al. (2012) compared the doubly bounded interpretations of scalar *Some*-sentences (derived via implicatures), as in *Some elephants are mammals*, with *Only*-variants such as *Only some elephants are mammals* [see Breheny et al. (2006) and Bale et al. (2011) for similar suggestions]. Their results were that the doubly bounded interpretations of *Some*-sentences are delayed relative to those of their *Only*-variants. Furthermore, participants (native speakers of English) were equally successful at verifying the doubly bounded interpretation whether it was derived via implicatures (i.e., *Some*-sentences) or semantically forced (i.e., *Only*-sentences). These findings indicate that the extra processing time observed in the generation of implicatures cannot be fully accounted for in terms of semantic complexity differences. They suggest rather that the additional cost required to arrive at the enriched interpretations of scalar sentences is specific to SI derivation, which reinforces previous evidence against the default model of SI.

### SIs and working memory: dual task results

Capitalizing on Bott and Noveck (2004) paradigm and results, De Neys and Schaeken (2007) investigated the *nature* of the cost associated with SIs (see also Dieussaert et al., 2011; Marty et al., 2013). Specifically, they tested the hypothesis that the cognitive operations underlying the generation of scalar inferences involve the central component of the working memory system, whose executive resources are well-known to play a substantial role in high-order cognition (e.g., Engle et al., 1999; Kane and Engle, 2002; Kane et al., 2004; De Neys, 2006; De Neys and Verschueren, 2006). Using a dual-task procedure, De Neys and Schaeken found that participants generated significantly less doubly bounded interpretations for scalar *Some*-sentences (e.g., *Some elephants are mammals*) when their executive working memory resources were tapped, while the same cognitive load did not interfere with their interpretation of equivalent *Some*-sentences when SIs did not matter (e.g., *Some mammals are elephants* is true no matter whether the SI is taken into account).

Importantly, the dual task results are independent from the processing results we mentioned before. Response times may reveal that doubly bounded interpretations are derived later than lower-bound interpretations, while dual-task studies may reveal what resources are involved in the derivation of doubly bounded interpretations, independent of when these interpretations are derived and accessed. It is useful to illustrate the contribution of dual task studies with the help of *only* sentences. (a) Assume that the output of Step 1 (see Table 2) is not normally accessible to introspective judgments. The interpretation that does not take into account the exclusion of alternatives is not considered as viable when *only* is present, but yet it has to be derived at some point in the computation. This situation aligns well with the description of the default model described for SIs: Step 2 *must* be undertaken after Step 1. (b) Assume, however, that central memory resources are involved at Step 2, i.e., in the derivation of doubly bounded interpretations of *only*. This could be the case for instance because Step 2 involves the derivation and comparison of alternatives. Assuming (a) and (b), we can see the distinct potential contributions of dual task and response time studies. Competing dual tasks may make the output of Step 1, i.e., the lower-bound interpretation, accessible; response times are unable to do so. In short, the output of Step 1 is in principle not accessible to introspection as a plausible, final interpretation for *only* sentences, but if the resources necessary to move to Step 2 are blocked, the lower-bound interpretation may become the only one that is accessible.

The pattern of results reported in De Neys and Schaeken (2007) suggests that the generation of an SI draws on memory resources. However, it does not determine which step in the derivation of an SI this additional memory effort should be attributed to, leaving open the question whether or not it is specific to SIs. Furthermore, the additional memory search necessary to verify doubly bounded propositions could have contributed to the extra memory resources needed to derive implicatures. That the greater complexity of these propositions has not been found to play a significant role in the delay of SI processing (cf. Bott et al., 2012) does not guarantee that it does not affect the cognitive resources needed to derive an SI in some way.

In the remainder of this article, we report on a dual-task experiment that addresses these two issues and refines the memory effect reported in De Neys and Schaeken (2007). First, our results provide further evidence that the memory tax incurred to SIs reflects a cognitive cost above the cost associated with the meaning complexity of doubly bounded sentences. Second, they suggest that this cognitive tax is more likely to be attributed to the *decision* to derive implicatures (cf. Table 1, Step 1), rather than to the computation of implicatures *per se* (cf. Table 1, Step 2). We will discuss how these results call for a closer investigation of the extent to which a silent operator like 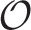 can be affected, like any disambiguation decision, by pragmatic considerations. We will also discuss how the present findings can be used as a baseline to investigate the exact nature of 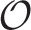 and contribute to the debate between the pragmatic and grammatical view on SIs.

Before going on, an important clarification is in order. There exist different perspectives on the nature, structure and functions of working memory, as can be seen in the diversity of theories that have been proposed (e.g., Baddeley and Hitch, 1974; Schneider and Detweiler, 1988; Just and Carpenter, 1992; Caplan and Waters, 1999; Cowan, 1999; Lovett et al., 1999; Baddeley and Hitch, 2000). Specifically, some researchers have emphasized the unitary nature of working memory (e.g., Engle et al., 1992, 1999; Miyake et al., 2001), whereas others have argued for a more domain-specific view and proposed that working memory consists of multiple separable subsystems (e.g., Daneman and Tardif, 1987; Just and Carpenter, 1992; Caplan and Waters, 1999; MacDonald and Christiansen, 2002). As Miyake and Shah (1999) observed, however, it is not always clear whether, despite their apparent opposition, these different perspectives are fundamentally incompatible or rather reflect differences in emphasis. The conceptualization of central memory resources adopted in this paper is liberally neutral with respect to this debate: it corresponds to the theoretical construct commonly used in cognitive psychology (cf. Baddeley and Hitch, 1974, 2000) to refer to the executive resources required by the memory system for controlling and coordinating the cognitive processes (e.g., retrieval strategies, task-shifting, etc.) responsible for the storage and manipulation of task-relevant information (visual or spatial) in the service of accomplishing a task. The main goal of our investigation is to determine the locus of the central memory cost in generating the SI from “some” to “not all” by using the comparison with “only.” Although the answer to this question is in large part orthogonal to the debate between the domain-general and the domain-specific view, we will point out in conclusion how our findings can be connected to existing assumptions about the structure of verbal working memory.

## Experiment

This experiment relies on the dual-task study conducted by De Neys and Schaeken (2007). It aims at comparing the relative memory demands on the derivation of the implicit doubly bounded meaning of the genuine scalar item *some* and on the computation of the explicit doubly bounded meaning of *only some*. Participants were asked to perform a truth-value judgment task *à la* Bott and Noveck (2004) on *Some*- and *Only*-sentences [cf. Bott et al., 2012, see (8) and (9) below], while they simultaneously had to remember a visual dot pattern (see Figure 2). The cognitive load on working memory was manipulated by varying the complexity of the to-be-memorized pattern, so that participants' memory resources were either minimally busy or more heavily tapped during the linguistic task. The rationale for this dual task procedure is that participants should not be able to appeal to their central memory resources for the linguistic task in conditions where the cognitive burden is high, i.e., in conditions where these resources are needed to perform the concurrent memory task. As a result, any process that also requires these resources should be impaired.

As we explained above, the derivation of an SI involves the following two steps: (1) the decision to derive the SI, and (2) more specific processes involving the derivation and comparison of alternatives, which processes are shared with the computation of the meaning of *only* (see Table 1). If the central memory resources are specifically required at this second step, which is at the core of what SIs are, then they should be required similarly in the processing of the semantic contribution of the grammatical operator *only*. Following these assumptions, any difference in the processing of the target *Some*- and *Only*-sentences would suggest that the memory effect occurs at another step of processing and, therefore, is not specific to the *derivation* of SIs, but rather to the *decision* to compute SIs.

### Participants

The participants were 16 native speakers of French (9 women), aged between 18 and 39 years, who were naive as to the purpose of the experiment. They participated in this study on a voluntary basis.

### Materials and tasks

#### Dot memory task

**Figure 1:**
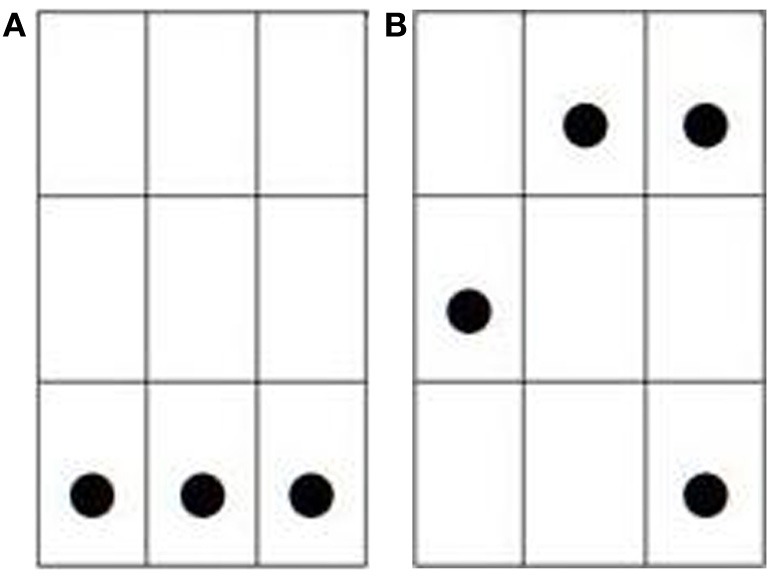
**Examples of dot patterns used in (A) LOW LOAD and (B) HIGH LOAD trials**.

The memory task was a short term storage task of visual patterns. These patterns consisted of a 3 × 3 matrix filled with 3 to 4 dots. As Bethell-Fox and Shepard (1988) showed, the cognitive effort required to encode such visual patterns increases with the stimulus complexity as measured, for instance, by the number of separated pieces that it contains. For the present study, the pattern complexity was manipulated by varying both the number and the arrangement of dots. The matrix was filled with 3 dots aligned horizontally or vertically (“one-piece” patterns) in the low load trials, and with 4 dots arranged in three separated pieces (“three-piece” patterns) in the high load trials, as exemplified in Figure 1. Following Miyake et al. (2001), the executive resources of working memory are tapped by the storage of such 4-dot patterns; alternatively, De Neys (2006) (see also De Neys and Verschueren, 2006) have observed that these resources are minimally burdened by the storage of such 3-dot patterns. To manipulate the load factor within subjects, participants were administered two blocks of trials: one block contained low load trials, and the other block contained high load trials.

#### Truth value judgment task

The linguistic task presented categorical sentences and asked participants to provide absolute truth-value judgments. Examples of the seven types of sentences used in this task are shown in Table 3. All the sentences were of the form “Q As are Bs,” where Q was one of the following quantifiers: *Some, Only some, All* (in French: *Certains, Seulement certains, Tous*)^4^.

**Table 3:** **Examples stimuli for the linguistic task**.

**Sentence type**	**Example**				**Correct response**
Some-total	Some	snakes	are	reptiles	T or F
Some-partial	Some	reptiles	are	snakes	T
Some-null	Some	snakes	are	flowers	F
Only-total	Only some	snakes	are	reptiles	F
Only-partial	Only some	reptiles	are	snakes	T
Only-null	Only some	snakes	are	flowers	F
All-total	All	snakes	are	reptiles	T

A and B were sets of individuals from a list of “categories” and “subcategories” (see Appendix). The set membership relationship between A and B was manipulated so that the inclusion of the A individuals in the B individuals was either *Total* (*A* ⊂ *B*, i.e., all As are Bs), *Partial* (*A* ∩ *B* ≠ ∅ and *A* ∩ *not(B)* ≠ ∅, i.e., some As are Bs and some As are not Bs) or *Null* (*A* ∩ *B* = ∅, i.e., no As are Bs). *Some-Total* sentences such as (8) correspond to the critical condition, as in Bott and Noveck (2004), because they have two potential interpretations depending on whether an SI is derived, and which interpretations have different truth-values^5^.

(8) Some snakes are reptiles.
       a. Lower-bound: Some (or all) snakes are reptiles.
       b. doubly bounded: Some but not all snakes are reptiles.

Basically, (8) is true under its lower-bound interpretation, but false under its doubly bounded interpretation with its *not-all* SI (since *all* snakes are reptiles). Hence, in a truth-value judgment task, participants' responses should indicate whether they went through the process of deriving an SI (“false” response) or whether they did not (“true” response). We were interested in comparing these sentences with *Only-Total* variants like (9), in which the addition of the word *only* turns the *not-all* component of the SI in (8) into a plain entailment of the sentence:

(9) Only some snakes are reptiles.
       a. Means: Some but not all snakes are reptiles.
       b. Cannot mean: Some snakes (or all) are reptiles.

Contrary to *Some-Total* sentences, *Only-Total* sentences are not ambiguous: (9) can only mean that *not all* snakes are reptiles. However, similarly as for *Some-Total* sentences, their doubly bounded interpretation requires the identification of a set of alternatives and the exclusion of stronger alternatives (see Table 1). Participants going through this process should therefore correctly judge these sentences as being false, but in circumstances in which this competition process is not manageable (e.g., because it requires memory resources that are busy) participants may judge these sentences as being true.

In addition to these target sentences, participants had to judge control sentences that were unambiguously true (i.e., *Some-Partial, Only-Partial* and *All-Total*) or unambiguously false (i.e., *Some-Null* and *Only-Null*). These sentences were added to ensure that participants would do the task appropriately and that the cognitive load would not interfere with the understanding of unproblematic sentences in general.

Eight lists of sentences were created using a Latin square design, so that every subcategory (cf. Appendix) was used only once per list. Each list was composed of 8 repetitions of the 3 *Total* conditions, and 2 repetitions of the 4 other conditions, giving a total of ((8 × 3) + (2 × 4)) = 32 sentences per list. Each participant was presented with two distinct lists of sentences: one list was used in the low load trials, and the other list in the high load trials.

### Procedure

**Figure 2:**
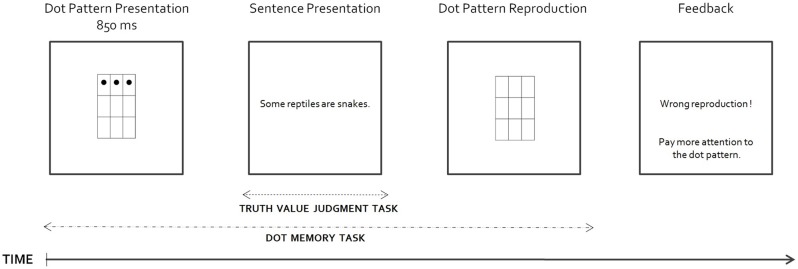
**General structure of a trial**.

Figure 2 illustrates the procedure. Each trial started with the brief presentation (850 ms) of a dot pattern in the center of a computer screen. The dot pattern was then replaced by a sentence, which remained on the computer screen until the participants provided a truth value judgment by pressing one of two keys (1 = false, 2 = true). Next, the participants had to reproduce the location of the dots in an empty matrix by using a numeric keypad. At the end of each trial, they received feedback on the quality of their pattern reproduction.

The participants were instructed that it was essential for the experiment to reproduce accurately the entire patterns of dots. With respect to the linguistic task, they were asked to read sentences and respond “true” or “false” according to whether the sentences were consistent with their general knowledge. The participants started with a short training composed of 4 complete trials (2 low load and 2 high load trials). The sentences used for the training were unrelated to the present experimental issue, and were simply included to help participants familiarize with the display. The participants were then administered two consecutive blocks of 32 sentences with a short break in between. For each participant, it was pseudo-randomly determined which type of trial blocks (low load or high load) they started with. In each block, items were presented in random order.

### Results

#### Dot memory task

**Figure 3:**
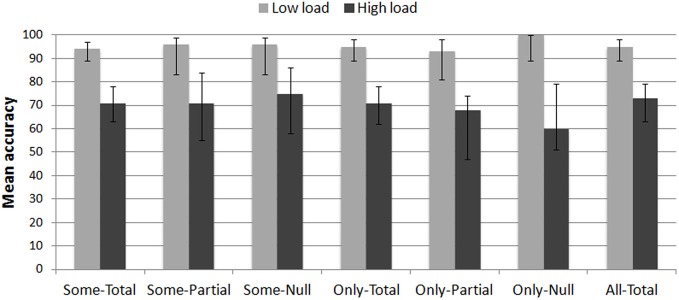
**Mean accuracy (in %) to the dot memory task in the LOW LOAD and HIGH LOAD trials as a function of the sentence type presented in the truth-value judgment task**. Error bars are 95% confidence intervals estimated from binomial distributions.

A response to the memory task was counted as accurate when the participant correctly reproduced the entire pattern of dots. Figure 3 reports the mean accuracy to the memory task in the low load and high load trials as a function of the sentence type participants were presented with for the truth-value judgment task.

Data were analyzed using a linear mixed-effects model (binomial family) predicting accuracy from Load and Sentence type. The model included random effects for Subject and Item, and random slopes for the interaction of the fixed effects grouped by Subject. Nested linear model comparisons were computed with the null model including the full random effect structure. There was a main effect of Load [χ^2^(1) = 43, *p* < 0.0001] such that the rate of accurate responses was significantly lower in the high load trials (*M* = 95%, CI_95%_ [93, 97]) than in the low load trials (*M* = 74%, CI_95%_ [69, 77]): β = 2.74, *z* = 4.2, *p* < 0.0001. No other effects or interactions were significant (all χ^2^ < 5.7, *p*s > 0.46). This first result confirms that 4-dot patterns were more demanding than the 3-dot patterns.

Responses to the memory task were analyzed further by examining dot recall performances when participants responded “false” to the target *Some-Total* (doubly bounded interpretations, with SIs) and *Only-Total* (correct doubly bounded interpretations) sentences. Data were fitted into a linear mixed-effects model predicting accuracy from Load and Target sentence^6^. Only the main effect of Load was significant (Load: β = 3.71, *z* = 4, *p* < 0.0001; Target: β = 0.76, *z* = 1.49, *p* = 0.2; Load × Target: β = 0.91, *z* = 0.69, *p* = 0.48). This second result ensures that there was no trade-off between dot recall performances and truth-value judgments to target sentences.

In subsequent analyses, trials for which participants did not accurately reproduce the entire pattern were removed (about 15% of the trials). According to a One-Way analysis of variance (anova), the mean number of removed trials did not significantly differ from one sentence type to another, in both low load and high load conditions (*F*s < 1, ns.).

#### Truth-value judgment task

Response times to the truth-value judgment task were analyzed to control for outliers. The data were approximately normally distributed for each subject and there were no obvious outliers (no datapoint was above or below two standard deviations from each subject's mean). Thus, no data points were removed on the basis of response times.

***Control sentences.*** Table 4 reports the mean accuracy scores to control sentences (with standard errors in parentheses).

**Table 4:** **Mean accuracy (in %) to the control sentences in the LOW LOAD and HIGH LOAD trials**.

**Sentence type**	**LOW LOAD**	**HIGH LOAD**
Some-partial	95 (4)	90 (5)
Some-null	96 (4)	100 (0)
Only-partial	100 (0)	96 (3)
Only-null	100 (0)	94 (4)
All-total	96 (2)	91 (3)

Standard errors are indicated in parentheses.

Performance was uniformly high, with a global mean score of 95% (CI_95%_ [92, 97]) in the low load trials, and of 94% (CI_95%_ [90, 97]) in the high load trials. Responses to control sentences were fitted into a linear mixed-effects model in a likelihood setting predicting accuracy from Load and Control type. The model included Subject and Item as random effects with random slopes for the interaction of the fixed effects grouped by Subject. Neither main effects, nor the interaction reached significance (Load: χ^2^(1) = 2.6, *p* = 0.1; Control: χ^2^(1) = 4.86, *p* = 0.3, Load×Control: χ^2^(1) = 0.58, *p* = 0.96). These control results confirm that participants did the task appropriately, and that the concurrent memorization of the complex 4-dot patterns did not interfere with their understanding of unproblematic sentences.

**Figure 4:**
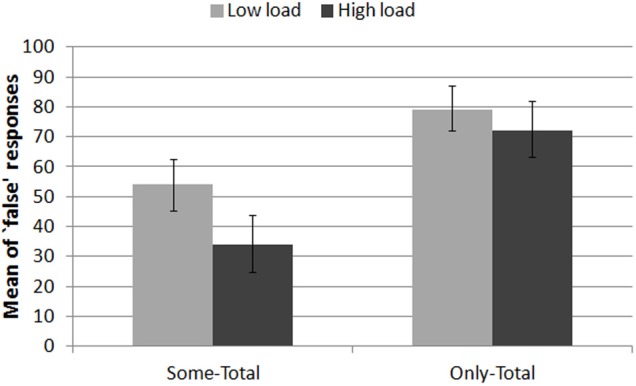
**Percentage of doubly bounded interpretations (“false” responses) to the target sentences in the LOW LOAD and HIGH LOAD trials**. Error bars are 95% confidence intervals estimated from binomial distributions.

***Target sentences.*** Figure 4 displays the percentage of “false” responses to *Some-Total* sentences and to *Only-Total* sentences, i.e., the rate of doubly bounded interpretations in both cases.

Participant's responses were fitted into a linear mixed-effects model that regressed the response variable against Load and Target type. The model included Subject and Item as random effects and random slopes for the interaction of the fixed effects grouped by Subject. Nested linear model comparisons were computed with the null model including the full random effect structure. There was a main effect of Load (χ^2^(1) = 7.33, *p* < 0.01), a main effect of Target (χ^2^(1) = 19.28, *p* < 0.0001) and a significant interaction between these two factors (χ^2^(1) = 5.07, *p* < 0.05).

*Post-hoc* analyses of the interaction between Load and Target were performed using multiple comparisons of means for general linear hypotheses (Tukey contrasts). They revealed that the Load effect was specific to *Some-Total* sentences. These sentences generated a significantly lower rate of “false” responses in the high load (*M* = 34%, CI_95%_ [25, 44]) than in the low load trials (*M* = 54%, CI_95%_ [46, 63]): β = −0.23, *z* = −3.44, *p* < 0.005. Alternatively, the rate of “false” responses to *Only-Total* sentences were about the same in both trial types: *M* = 72% (CI_95%_ [64, 79]) vs. *M* = 79%(CI_95%_ [72, 87]), β = −0.01, *z* = −0.29, *p* = 0.99^7^.

Hence, the present results show that working memory is not recruited similarly in the computation of the *not-all* component of meaning of sentences with *only* as it is recruited in the derivation of SIs: while less doubly bounded interpretations were derived for the SI-sentences under high cognitive load, the concurrent memorization of the 4-dot patterns did not substantially interfere with the interpretation of their *Only*-variants.

## Discussion

In this article, we have discussed two possibilities for localizing the additional memory demands associated with doubly bounded interpretations of scalar sentences like *Some snakes are reptiles*. One possibility referred to the processing step involving the decision to draw the *not-all* implicature beyond the basic meaning of the implicature trigger (*some or all*). The other possibility was that the demands are linked to the subsequent interpretative step in which comprehenders compute the enriched, doubly bounded interpretation (which includes the SI), that requires them to identify the relevant alternatives and deny the stronger ones(i.e., not all). On this latter account, the demands on memory should be equivalent when processing sentences like *Only some snakes are reptiles*, whose doubly bounded interpretations are made explicit by the presence of the overt lexical item *only*. To test this alternative, we used a dual-task paradigm similar to De Neys and Schaeken (2007).

First, we replicated De Neys and Schaeken's results showing that implicit doubly bounded interpretations attached to the word *some* come at an extra memory cost. Second, we found no evidence for a similar memory cost in the derivation of comparable (although explicit) doubly bounded interpretations associated with the phrase *only some*. Specifically, tapping participants' executive resources induced a significant decrease of *not-all* implicatures associated with *some*, and no comparable decrease of *not-all* inferences associated with *only*. These findings make two contributions. They establish that the memory cost occurring in the processing of SIs is above that associated with the *verification* of upper-bound interpretations, and has to be attributed to semantic/pragmatic *computations*. Furthermore, they suggest that the executive working memory resources are not required at the step of processing which is at the core of what SIs are, because this subprocess, which involves comparison and falsification of stronger alternatives, is also necessary to compute the *not-all* inferences associated with *only*. Hence, the extra memory cost incurred to the processing of an SI is due to the *decision* to derive it, rather than to its derivation *per se*.

An important issue that we would like to address concerns the generality of these findings: how would the present results generalize across conversational situations? Indeed, as most experimental investigations on semantic-pragmatic phenomena, our study can be said to exhibit some level of unnaturalness, for example because stimulus sentences were divorced from explicit communicative goals. Therefore, the paradigm we used might have disfavored pragmatic, doubly bounded interpretations, leading participants to focus instead on the logical aspects of sentence meanings. We may thus wonder whether the observed memory effect would get reduced—or even disappear—in a more naturalistic conversational setting where doubly bounded interpretations are contextually supported. As a point of comparison, researches on syntactic ambiguity have shown that the availability of contextual information can influence the processing of words in sentences and facilitate ambiguity resolution (Ferreira and Clifton, 1986; Clifton and Ferreira, 1989; Spivey-Knowlton et al., 1993; MacDonald et al., 1994; Trueswell and Tanenhaus, 1994; Spivey and Tanenhaus, 1998, *inter alia*)^8^. Future investigations would be needed to determine whether such context effects can affect the weight of the memory cost observed in the processing of a SI. Precisely, following the present results, the hypothesis would be that the memory effect should be reduced when contextual information facilitates the decision step, for instance by biasing comprehenders to the pragmatic interpretations. Such results would further support the idea that the processing principles that account for ambiguity resolution are not specific to language at all, but rather reflect general properties (e.g., properties of memory) that are involved in non-linguistic domains such as decision making.

From a more theoretical perspective, we discussed recent grammatical views on SIs that have pointed towards a close analogy between overt *only* and a covert operator 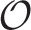. Our results show that overt *only* and SIs behave differently in terms of memory demands. Furthermore, the fact that the complexity of a visual memory task affects the decision to derive SIs suggests that this stage of processing is employing domain-general executive mechanisms (Novick et al., 2005). Under the additional plausible assumption that the processes involved in interpretive processing are distinct from those involved in other verbally mediated functions such as reasoning procedures (e.g., Caplan and Waters, 1995, 1999; Waters and Caplan, 2001), one could be thus tempted to go one step forward, and conclude that the present results provide evidence against the grammatical approach to SIs. For, the computations of a purely grammatical exhaustivity operator should take place within the linguistic system, and therefore not be tapping into a domain-general resource pool. We would like, however, to invite our readers to not commit themselves to such a conclusion which, according to us, relies on a false dichotomy regarding the question of modularity in the debate between the Gricean and the grammatical approach.

Precisely, as we emphasized earlier, the Gricean picture does not have a greater monopoly on domain-general reasoning procedures than the grammatical view on domain-specific computations. Both approaches do involve interactions between grammar and pragmatics at all relevant stages of implicature computation. The memory cost in generating SIs may be in fact rooted in the optionality of the 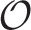 operator, that is in the decision to apply or not the operator, and thus remains compatible with the grammatical view. For, the decision to apply 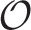 is a pragmatic decision, even in a grammatical approach of SIs (just like any disambiguation decision is pragmatic), and it is at this pragmatic level of sentence comprehension that the investigated memory cost seems to be observed. Thus, the present results cannot be used to tease apart the Gricean and the grammatical approaches, but they can be used as a baseline for future investigations. In principle, one could investigate whether the behavioral properties of SIs (e.g., extra processing time, memory cost) can be reproduced with other linguistic operators that would be covert and optional, and less controversially either syntactic or pragmatic [covert *even* may be such an operator, see (Heim, 1984; Krifka, 1995; Chierchia, 2006; Charnavel, 2012a,b)], and use these results to draw conclusions about the nature of 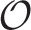.

### Conflict of interest statement

The authors declare that the research was conducted in the absence of any commercial or financial relationships that could be construed as a potential conflict of interest.

## Footnotes

^1^For the sake of simplicity and to make sure we will have good control of the analysis of our examples, we will mainly focus on the implicatures generated by the use of the quantifier *some*, which scalar behavior has been documented in various languages of the world. However, scalar inferences are triggered by numerous phrases and scales such as connectives (e.g., 〈or, and〉), modals (e.g., 〈might, must〉) or gradable adjectives (e.g., 〈good, excellent〉) (Horn, 1972; Hirschberg, 1991).

^2^Note, however, that the processing stage at which the decision step occurs, as well as the psycholinguistic reality it covers, may slightly differ depending on the view on sentence ambiguity resolution one adopts. To illustrate this point, let us see how it can be integrated with two possible views corresponding loosely to serial and parallel processing. Following a serial parsing view, the parser is assumed to entertain only one reading at a time. If that reading is perceived as incorrect or unsatisfying, then it may be abandoned and the sentence is re-parsed. In the case of scalar sentences, the decision of applying  corresponds to the decision to look forward to accessing a second reading if the literal reading is not satisfying (e.g., because the speaker is expected to be very cooperative). Under this view, the decision step does not require the strengthened reading to be already available (and the alternatives to be calculated). In response to a reviewer's comment, note that accessing the result of the -application is not necessary in order to know whether we are satisfied with the literal reading (even though, once the result is obtained, one may still have the option of discarding this option and look for other options). By contrast, following a parallel parsing view, the parser entertains all possible readings simultaneously and, as more evidence is encountered, the unintended readings are eliminated leaving only the intended one. Nonetheless, one needs to decide which set of readings to entertain. In the case of scalar sentences, the decision to apply  corresponds to the decision to enlarge the set of possible readings and generate an alternative reading to be compared to the literal one. Expectations are therefore the same as in the previous approach. If the memory cost occurs when deciding which alternative reading is included in the set of candidates, then comprehenders may not add the enriched meaning in the set of candidate meanings (and therefore they will have no chance to select that reading) if their memory resources are tapped.

^3^As a reviewer pointed out, the time course of implicature generation has varied from one study to another [see Breheny et al. (2013) for relevant discussion]. Some studies have found that accessing the doubly-bounded reading of “some” comes at a delay relative to the lower-bound reading (cf. Bott and Noveck, 2004; Huang and Snedeker, 2009). These delayed effects have generally been interpreted as showing that lower-bound readings are accessed earlier than doubly bounded readings. Nonetheless, other studies have suggested that such a conclusion might be too strong (Sedivy, 2003; Degen and Tanenhaus, 2011; Grodner and Sedivy, 2011; Breheny et al., 2013): although implicatures may be accessed at a delay, they do not have to be. In particular, Degen and Tanenhaus (2011) have shown that hearers are able to compute implicatures as rapidly as literal content under the right circumstances (i.e., when the set of relevant natural alternatives is reduced).

^4^Just like its English counterpart *some*, the quantifier *certains* can syntactically combine with an NP to form (7-a) a partitive or (7-b) a non-partitive construction:

(7) a. Certains des reptiles sont des serpents.
          “Some of the reptiles are snakes.”
       b. Certains reptiles sont des serpents.
          “Some reptiles are snakes.”

Degen and Tanenhaus (2011) have shown that the syntactic form of the *some*-NP affects the processing of SIs. In a nutshell, partitive “some of the As” has been found to provide comprehenders with a better cue to an implicature than non-partitive “some As”. The present paper does not address this issue. Sentences in our experiment involved only non-partitive nominal constructions as shown in (4). The non-partitive construction was equally used when *only* was present (the partitive construction is also an option with *only*).

^5^A sentence such as “Some snakes are reptiles” should normally sound odd because it can trigger the SI that not all snakes are reptiles, which mismatches the piece of common knowledge that all snakes are reptiles. The oddness of such sentences is part of the trick that originates in Bott and Noveck (2004), and has been theoretically investigated in Magri (2009, 2011) as a diagnostic for SIs.

^6^The model also included a random effect for Subject, and random slopes for the interaction of Load and Target grouped by Subject.

^8^Thanks to a reviewer for suggesting this parallel to us.

## Appendix

### List of categories and subcategories

Categories and subcategories used in the linguistic task are given in Table A1 (actual items were in French). *Some-Null* (e.g., Some snakes are flowers) and *Only-Null* (e.g., Only some snakes are flowers) sentences were constructed by systematically pairing subcategories relative to animals with (wrong) categories relative to plants, and vice-versa.

**Table A1:** **Categories and subcategories used in the linguistic task (English translation, actual items were in French)**.

**Categories**	**Subcategories**			
Flower	Daisy	Tulip	Poppy	Daffodil
Fruit	Lemon	Banana	Strawberry	Orange
Tree	Poplar	Fir	Apple tree	Beech
Vegetable	Leek	Spinach	Broccoli	Carrot
Bird	Sparrow	Gull	Crow	Parrot
Fish	Trout	Salmon	Tuna	Carp
Reptile	Crocodile	Snake	Lizard	Alligator
Insect	Ant	Mosquito	Wasp	Fly
